# Kiperin Postbiotic Supplement-Enhanced Bacterial Supernatants Promote Fibroblast Function: Implications for Regenerative Medicine

**DOI:** 10.3390/biomedicines13061430

**Published:** 2025-06-10

**Authors:** Lutfiye Karcioglu Batur, Cuneyd Yavas, Yağmur Ekenoğlu Merdan, Ashabil Aygan

**Affiliations:** 1 Department of Molecular Biology and Genetics, Faculty of Engineering and Natural Sciences, Biruni University, 34015 Istanbul, Turkey; 2 Biruni University Research Center (B@MER), Biruni University, 34015 Istanbul, Turkey; 3 Department of Medical Microbiology, Faculty of Medicine, Biruni University, 34015 Istanbul, Turkey; 4 Department of Biology, Faculty of Science, Kahramanmaras Sutcu Imam University, 46050 Kahramanmaras, Turkey

**Keywords:** postbiotic, paraprobiotic, fibroblast, tissue regeneration, smart supplement

## Abstract

**Background/Objectives:** Kiperin Postbiotics, defined as non-viable metabolic products derived from probiotics, have gained attention as potential modulators of cellular responses involved in tissue repair. This study aimed to evaluate the effects of a postbiotic supplement (PS)—composed of inactivated strains of *Escherichia coli*, *Lacticaseibacillus rhamnosus*, and *Lactiplantibacillus plantarum*—on fibroblast function, particularly in the context of bacterial secretomes from common pathogenic strains. **Methods:** Human fibroblast cell lines (HFF-1 and CCD-18Co) were treated with cell-free supernatants (CFS) from *E. coli* ATCC 25922, *Staphylococcus aureus* ATCC 29213, and *Enterococcus faecalis* ATCC 29212, either alone or in combination with the PS. Assessments included cell count, migration (via scratch assay), oxidative stress levels, and expression of immune-related genes (*IL-6*, *IL-10*, *TNF-α*, *DRD4*). **Results:** CFS from *E. faecalis* significantly increased fibroblast counts, whereas *E. coli* and *S. aureus* CFS reduced cell counts and elevated oxidative stress. Co-treatment with PS reversed these effects in a strain-dependent manner by lowering oxidative stress and partially restoring cell proliferation. Scratch assays demonstrated enhanced migration in PS-treated fibroblasts. Gene expression analyses revealed no statistically significant changes, though variable trends were observed across treatment groups. **Conclusions:** PS may mitigate the harmful effects of certain bacterial secretomes while preserving or enhancing beneficial ones. Its ability to reduce oxidative stress and promote fibroblast proliferation and migration suggests a potential pro-regenerative role in vitro. Although gene expression changes were limited, the results offer initial insights into the underlying molecular responses influenced by postbiotic supplementation.

## 1. Introduction

The application of probiotics in promoting tissue health has garnered significant attention, particularly concerning their role in modulating fibroblast activity [1,2]. Fibroblasts are pivotal in maintaining the structural integrity of connective tissues and play a crucial role in wound healing through collagen synthesis and extracellular matrix production [3]. While the beneficial effects of live probiotics on fibroblasts have been well-documented, recent studies have explored the potential of inactivated probiotic microorganisms, commonly referred to as paraprobiotics [4], in influencing fibroblast function [2,5,6].

Paraprobiotics are non-viable microbial cells that, despite being inactivated, retain their ability to confer health benefits to the host [4]. These benefits are primarily attributed to the bioactive components present in the inactivated cells, such as cell wall fragments, metabolites, and nucleic acids, which can interact with host cells to elicit physiological responses [7]. The use of paraprobiotics offers advantages, including improved safety profiles, especially in immunocompromised individuals, and enhanced stability in product formulations [8].

Research has demonstrated that inactivated probiotic strains can positively influence fibroblast activity. For instance, metabolites derived from *Bifidobacterium lactis* Bl-04^®^ have been shown to promote dermal fibroblast proliferation and confluence, as well as balance collagen homeostasis under inflammatory conditions. These findings suggest potential anti-aging effects on skin structure [9]. Additionally, studies have indicated that both live and heat-killed *Lactiplantibacillus plantarum* can enhance fibroblast migration and collagen synthesis, thereby accelerating wound healing processes [1]. The mechanisms underlying these effects involve the interaction of paraprobiotic components with fibroblast surface receptors, leading to the activation of signaling pathways that regulate cell proliferation, migration, and extracellular matrix production. Moreover, paraprobiotics can modulate the local immune response by influencing cytokine production, thereby creating a conducive environment for tissue repair and regeneration [4].

The postbiotic formulation used in this study, Kiperin^®^ Postbiotic 210 Billion CFU *Rhamnosus* & *Plantarum* Digestion (700 mg), is a commercially available preparation designed to support gastrointestinal health. Each capsule contains heat-inactivated *Lacticaseibacillus rhamnosus* and *Lactiplantibacillus plantarum* strains, standardized to a viable equivalent of 10 billion CFU per strain before inactivation. This product is one of the selected formulations within the Kiperin^®^ brand and is certified under the “Smart Food Supplement” quality assurance program. The selection of this specific product was based on its defined strain composition and manufacturing process, which aligned with the experimental objectives of evaluating postbiotic effects on fibroblast function.

Cell-free supernatants (CFS) derived from probiotic or commensal bacteria contain a complex mixture of metabolites, peptides, short-chain fatty acids, and signaling molecules that can influence host cell behavior [10]. These bioactive components may stimulate fibroblast proliferation by modulating redox balance, enhancing mitochondrial activity, and activating growth-related pathways such as MAPK/ERK and PI3K/AKT signaling [11,12].

For instance, certain bacterial metabolites have been shown to reduce oxidative stress and promote extracellular matrix remodeling, thereby creating a more favorable microenvironment for fibroblast growth and function [13]. Additionally, studies have demonstrated that microbial-derived compounds can influence fibroblast behavior through various signaling pathways, contributing to tissue repair and regeneration [14]. In this study, the potential effects of a postbiotic supplement (PS) which contains inactivated probiotic microorganisms in powdered form, including *Lacticaseibacillus rhamnosus* and *Lactiplantibacillus plantarum* on CFS produced by microorganisms commonly found in the gut microbiota (*Escherichia coli* ATCC 25922, *Staphylococcus aureus* ATCC 29213, and *Enterococcus faecalis* ATCC 29212), were investigated. For this purpose, the effects of CFS obtained from bacterial strains grown in media with and without PS were compared in terms of their impact on two fibroblast cell lines, HFF-1 and CCD-18Co cells.

## 2. Methods

### 2.1. Cell Culture

The study protocol was approved by the Scientific Research Ethics Committee of Biruni University on 12 December 2024 (Approval No: 2024-BIAEK/05-31). HFF-1 fibroblast cells isolated from the foreskin were obtained from ATCC (SCRC-1041), and CCD-18Co human colon fibroblasts cells which were obtained from ATCC (CRL-1459).

All cell lines were cultured in Dulbecco’s Modified Eagle Medium (DMEM) supplemented with 10% fetal bovine serum (FBS) and 1% penicillin–streptomycin antibiotics at 37 °C in a humidified incubator containing 5% CO_2_ (Kabin Incubator). The cells were maintained in these conditions throughout the experiments.

Kiperin^®^ PS (Istanbul, Turkiye) is a powdered formulation containing inactivated probiotic microorganisms, specifically *Lacticaseibacillus rhamnosus* and *Lactiplantibacillus plantarum*, each at a concentration of 5 billion CFU/day (5 × 10^9^ CFU/day) per capsule. To determine the optimal in vitro dose, CCD-18Co and HFF-1 fibroblast cell lines were treated with four different concentrations of PS (31.2 µg/mL, 62.4 µg/mL, 83.2 µg/mL, and 114 µg/mL). The fibroblast cells were counted in triplicate using a Mindray BC-6800 (Shenzhen, China) automated cell counter. According to the results, the most effective concentration was determined as 83.2 µg/mL for CCD-18Co cells and 31.2 µg/mL for HFF-1 cells. In parallel, cell-free supernatants (CFS) derived from *Escherichia coli*, *Staphylococcus aureus*, and *Enterococcus faecalis* were applied to both fibroblast cell lines. The experimental design consisted of nine groups, categorized based on the application of each bacterial supernatant either alone or in combination with Kiperin^®^ PS:

1. Negative control: HFF-1 and CCD-18Co cells were cultured in standard growth medium without any treatment. This group served as a baseline for comparison.
2. Positive control treated with H_2_O_2_: cells were treated with H_2_O_2_, which is a known stimulatory agent for oxidative stress, to evaluate its effects in comparison to bacterial supernatants.
3. Directly PS-treated cells: cells were treated directly with 24 mg/mL PS for 48 h.
4. *S. aureus* control: cells were treated with CFS obtained from *S. aureus* ATCC cultures without PS for 48 h. This group assessed the baseline effect of *S. aureus*-derived CFS.
5. *E. faecalis* control: cells were treated with CFS from *E. faecalis* cultures grown without PS for 48 h. This group evaluated the effects of *E. faecalis* CFS.
6. *E. coli* control: cells were treated with CFS from *E. coli* cultures grown without PS for 48 h. This group assessed the impact of *E. coli* CFS.
7. *S. aureus* treated with PS: cells were exposed to CFS obtained from *S. aureus* cultured in a medium supplemented with PS for 48 h. This group analyzed the impact of postbiotic-modified *S. aureus* supernatant.
8. *E. faecalis* treated with PS: cells were treated with CFS from *E. faecalis* cultures grown in a medium supplemented with PS for 48 h. This group examined the effect of postbiotic-treated *E. faecalis* CFS.
9. *E. coli* treated with PS: cells were treated with CFS from *E. coli* cultures grown in a medium supplemented with PS for 48 h. This group assessed how PS alters the effects of *E. coli* supernatant on fibroblast cells.

All PS treatments in bacteria were for 48 h.

### 2.2. CFS Preparation

To prepare the CFS, standard strains commonly found in the gut microbiota and present in our culture collection were used: *Escherichia coli* ATCC 25922, *Staphylococcus aureus* ATCC 29213, and *Enterococcus faecalis* ATCC 29212. Each standard strain was incubated in brain heart infusion (BHI) broth at 37 °C for 24 h. Following incubation, 100 µL of the 24 h liquid culture was taken and inoculated onto BHI agar plates, which were then incubated at 37 °C for another 24 h.

From the pure standard strain colonies grown on the BHI agar plates, several colonies were selected and suspended in BHI broth containing 24 mg/mL PS. As a control group, inoculation was performed in BHI broth without PS. These suspensions were incubated at 37 °C for 24 h. After incubation, 5 mL of the standard strain culture was collected and transferred to sterile centrifuge tubes. The samples were centrifuged at 3000 rpm for 10 min to remove cellular debris. The supernatant was carefully collected and passed through a 0.22 µm pore-size filter (Thermofisher Merck Millex™-GS Sterile Syringe Göteborg, Sweden) for sterilization before being transferred to a sterile tube.

The CFS preparations were sterilized and applied to fibroblast cells cultured in 6-well plates containing DMEM supplemented with 10% FBS. Each well received 2 µL of CFS, resulting in a final dilution ratio of 1:1000 (*v*/*v*). At this dilution, the residual BHI content was minimal and did not alter the osmolarity or nutritional profile of the eukaryotic culture medium.

### 2.3. Method of Obtaining Postbiotics

The postbiotics used in this study were obtained by heat inactivation of *Lactiplantibacillus plantarum* and *Lacticaseibacillus rhamnosus* strains. These bacterial strains were obtained from commercial probiotic preparations and incubated in Man–Rogosa–Sharpe (MRS) medium under aerobic conditions at 37 °C with 120 rpm shaking for 10–14 h to reach the logarithmic growth phase. At the end of this period, the cultures were centrifuged at 5000× *g* for 10 min, the cells were collected and washed with phosphate-buffered saline (PBS). The resulting cell suspension was standardized to 1 × 10^10^ CFU/mL and incubated at 90 °C for 2 h. After heat treatment, viability control was performed by surface cultivation on MRS agar; no colony formation was observed, confirming complete inactivation. Cell integrity was confirmed by Gram staining.

The thermal inactivation process eliminates the viability of probiotic cells but preserves biologically active molecules such as surface structures and cell wall components. Therefore, although heat-inactivated cells do not contain live microorganisms, they can maintain their immunomodulatory effects and are considered as postbiotics. This process provides significant advantages not only in terms of microbial safety, but also in terms of stability, shelf life, and ease of application.

The production process was carried out with a defined and reproducible technological approach, and in accordance with the postbiotic concept, bacterial preparations that do not have viability but provide biological benefits on the host were obtained. In this context, this heat-inactivated *L. plantarum* and *L. rhamnosus* fractions have been put into use as safe and effective postbiotic components (Table 1).

### 2.4. Cell Scratch Assay

To evaluate cell migration, a cell scratch assay was performed. At least 90% of the confluent monolayers of cells were scratched with a sterile pipette tip, creating a uniform gap. After creating the scratch using a sterile pipette tip, the culture medium was immediately replaced, and the wells were gently washed with phosphate-buffered saline (PBS) to remove any detached or dead cells. This ensured that only viable, adherent cells remained in the culture for subsequent imaging and analysis. It is also important to note that, following confluence and mechanical disruption, cells at the scratch margins may adopt a more compact or spherical morphology as they reorient and begin migrating into the wound area. This morphological change is typical of the early wound healing response. Non-viable cells, being unable to attach, were excluded from imaging fields during microscopy. Therefore, the cells observed at the wound edges represent viable cells actively participating in the migration process.

Images of wound closure of HFF-1 and CCD-18Co cells were taken at 0, 6th, and 20th hours, using an inverted microscope at the same marked position. The migration rate was quantified by measuring the percentage of cellular closure over time according to the literature [15]. Specifically, the wound area was measured at 0 h (baseline) and at subsequent time points (6 h and 20 h), and the migration rate was calculated using the formula:

Migration rate (%) = [(Area_0h_ − Area_t_)/Area_0h_] × 100

where Area_0h_ is the scratch area at the initial time point (0 h) and Area_t_ is the scratch area at the specified time (t). This formula reflects the proportion of the wound area that was closed by migrating cells. All measurements were performed using standardized image analysis software to ensure consistency.

### 2.5. Measurement of Oxidative Stress Levels and Antioxidant Capacity

Cell lysates were prepared from HFF-1 and CCD-18Co cell lines following standard protocols and stored at −80 °C until analysis. All reagents and samples were equilibrated to room temperature before use.

The total oxidant status (TOS) and total antioxidant status (TAS) levels in three cell lines were measured using commercial assay kits provided by Rel Assay Diagnostics (Mega Tıp San. Tic. Ltd. Sti., Gaziantep, Turkey). Both assays were performed according to the manufacturer’s protocol, utilizing a spectrophotometer (Epoch Microplate Spectrophotometer, BioTek Instruments, Winooski, VT, USA) to quantify the absorbance changes associated with the oxidative and antioxidative properties of the samples.

To measure TOS, the assay relied on the oxidation of ferrous ions to ferric ions by oxidant molecules in the sample. The ferric ions formed a colored complex with a chromogen in an acidic medium, and the color intensity was proportional to the total oxidant molecules present. For the assay, 45 µL of cell lysate, standard solution, or distilled water (used as a blank) was mixed with 300 µL of buffer solution (pH 1.75). The absorbance of the reaction mixture was first read at 530 nm after 30 s. Subsequently, 15 µL of ferrous ion solution was added to the mixture, and after incubation for 5 min at 37 °C or 10 min at room temperature, the absorbance was read again. The total oxidant status was calculated based on the change in absorbance and expressed as micromolar hydrogen peroxide equivalents per liter (µmol H_2_O_2_: equiv/L).

For TAS measurements, antioxidants in the sample reduced dark blue–green ABTS radical cations to their colorless form. The assay used 18 µL of cell lysate, standard solution, or distilled water (blank), which was mixed with 300 µL of acetate buffer (pH 5.8). The initial absorbance of the reaction mixture was measured at 660 nm after 30 s. Subsequently, 45 µL of ABTS prochromogen solution was added, and after incubation for 5 min at 37 °C or 10 min at room temperature, the final absorbance was recorded. The antioxidant capacity was quantified based on the change in absorbance and expressed as Trolox equivalents per liter (mmol Trolox equiv/L).

The TOS and TAS levels were calculated and analyzed statistically, with results expressed as mean ± standard deviation (SD).

### 2.6. RT-qPCR for Apoptotic and Cell Cycle Regulatory Markers Expression

Total RNA was extracted from HFF-1 and CCD-18Co cells, and the concentration was determined using a NanoDrop (ThermoFisher, Waltham, MA, USA). Subsequently, 500 ng of RNA was reverse-transcribed into complementary DNA (cDNA) using the OneScript^®^ Plus cDNA Synthesis Kit (Applied Biological Materials Inc., Richmond, BC, Canada) according to the manufacturer’s instructions. The reverse transcription reaction was carried out with Moloney murine leukemia virus reverse transcriptase.

In this study, the selected genes represent both classical inflammatory markers and genes potentially involved in immune regulation. *TNF-α* (tumor necrosis factor-alpha) and *IL-6* (interleukin-6) are key pro-inflammatory cytokines that play central roles in the acute phase response and cytokine signaling. *IL-10* (interleukin-10), on the other hand, is an anti-inflammatory cytokine that contributes significantly to the modulation and resolution of immune responses. These three cytokines were chosen as primary indicators for evaluating the inflammatory balance. *DRD4* (dopamine receptor D4 gene) was included as an exploratory marker due to its potential immunomodulatory role through dopaminergic signaling pathways. Emerging literature suggests that dopamine receptors may influence immune cell function and that dopaminergic signaling could play a role in neuroimmune interactions and inflammation. Thus, *DRD4* was incorporated to explore the possible broader cellular effects of the treatments beyond classical inflammatory pathways.

Quantitative real-time PCR (RT-qPCR) was performed using the BlasTaq™ 2X qPCR MasterMix (Applied Biological Materials Inc., Richmond, BC, Canada) following the supplier’s protocol. Each reaction contained 2 µg of cDNA template, 1 µM of each primer, and the appropriate volume of MasterMix, adjusted to a final volume of 25 µL with nuclease-free water. The primer sequences used in this study were designed using the Primer-BLAST tool (NCBI) based on the reference sequences of the target genes obtained from the GenBank database. The specificity of the primers was first evaluated through in silico analysis to minimize the possibility of off-target binding. For experimental validation, PCR amplification was performed, followed by agarose gel electrophoresis. The observation of a single band of the expected size confirmed that each primer pair was specific and effective. The primers used were as follows: for *TNF-α*, forward 5′-CAGCCTCTTCTCCTTCCTGAT-3′ and reverse 5′-GCCAGAGGGCTGATTAGAGA-3′; for *IL-6*, forward 5′-GGCACTGGCAGAAAACAACC-3′ and reverse 5′-AGCTTGGGGCATCACCTCCT-3′; for *IL-10*, forward 5′-CCAGGGCACCCAGTCTGAGA-3′ and reverse 5′-CCAGGAGAAATCAAACAGAG-3′; for *DRD4*, forward 5′-TCGTCTACTCCGAGGTCCAG-3′ and reverse 5′-CGAACCTGTCCACGCTGAT-3′; for *GAPDH* (housekeeping gene), forward 5′-CCACCCATGGCAAATTCC-3′ and reverse 5′-TGGGATTTCCATTGATGACAAG-3′.

Amplification and detection were conducted on a Bio-Rad CFX96™ Real-Time PCR System (Hercules, CA, USA) under the following cycling conditions: initial denaturation at 95 °C for 3 min, followed by 40 cycles of denaturation at 95 °C for 15 s, annealing at 60 °C for 15 s, and extension at 72 °C for 15 s. Fluorescence data were collected at the end of each extension step. Relative gene expression levels were calculated using the ΔΔCt method, normalizing the gene expressions to *GAPDH* as the internal control.

### 2.7. Statistical Analysis

All statistical analyses were conducted using GraphPad InStat 3.1 software (San Diego, CA, USA). The distribution of the data was assessed for normality using the Kolmogorov–Smirnov test. For data that followed a normal distribution, comparisons between the two groups were made using the unpaired *t*-test and ones between three or more groups were made using one-way analysis of variance (ANOVA), followed by Tukey’s post hoc test for pairwise comparisons. For non-normally distributed data of two groups, the Mann–Whitney U test was applied. For non-normally distributed data of three or more groups, the Kruskal–Wallis H test was used, and Dunn’s post hoc test was applied to identify differences between specific groups. A *p*-value of less than 0.05 was considered statistically significant for all analyses. While exact *p*-values were reported in the text to reflect the strength of significance, the cut-off for statistical significance remained consistently set at *p* < 0.05 throughout the study.

## 3. Results

### 3.1. Effects of Postbiotic and Bacterial CFS Treatments on Fibroblast Counts

**Figure 1:**
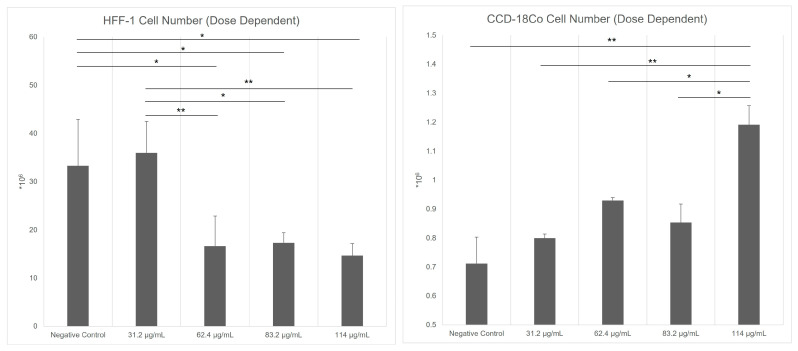
Effects of direct postbiotic supplement (DPS) on HFF-1 and CCD-18Co cell number. HFF-1 fibroblast cells were treated with/without five different amounts of DPS directly. Cell numbers were quantified after 48 h of treatment. Data are expressed as mean ± standard deviation (SD). Statistical significance level was determined as 0.0012 for HFF-1 and 0.003 for CCD-18Co. * *p* < 0.05, ** *p* < 0.01.

**Figure 2:**
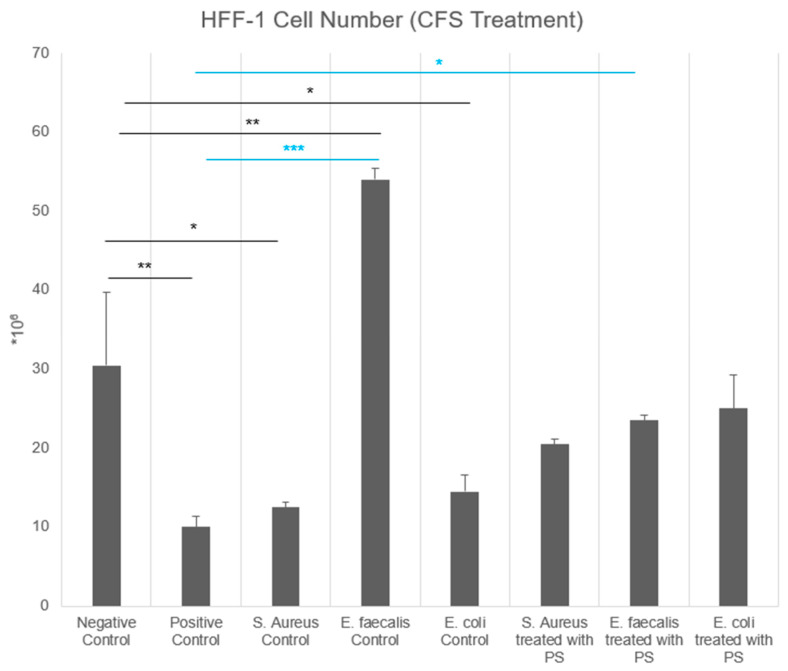
Effect of postbiotic supplement (PS) and bacterial cell-free supernatants (CFS) on HFF-1 fibroblast cell number. HFF-1 fibroblast cells were treated with/without CFS obtained from *Staphylococcus aureus*, *Enterococcus faecalis*, and *Escherichia coli* cultured in either standard BHI broth or BHI broth supplemented with postbiotic supplement (PS). Cell numbers were quantified after 48 h of treatment. Data are expressed as mean ± standard deviation (SD). Statistical significance level was determined as <0.0001. * *p* < 0.05, ** *p* < 0.01, and *** *p* < 0.001.

The effects of four different doses of direct PS (DPS) on HFF-1 fibroblast cell number were analyzed and the safe dose of PS on these cells was determined as 31.2 µg/mL (Figure 1). The effects of four doses of DPS on HFF-1 fibroblast cell number were analyzed, and the safe dose of PS on these cells was determined as 31.2 µg/mL (Figure 1). On the other hand, the effects of bacterial CFS on HFF-1 fibroblast cell number were analyzed, and significant differences were observed between experimental groups (*p* < 0.0001) (Figure 2). The positive control group exhibited a significant reduction in cell number compared to the negative control (*p* < 0.01). Similarly, the *S. aureus* control (*p* < 0.05) and *E. coli* control (*p* < 0.05) groups demonstrated significantly lower cell numbers than the negative control, indicating a suppressive effect of their respective CFS. In contrast, the *E. faecalis* control group showed a markedly higher fibroblast count compared to all other groups (*p* < 0.001), suggesting a proliferative effect of its CFS.

Treatment with PS modulated these effects (Figure 2). While *S. aureus* CFS with PS and *E. coli* CFS with PS did not significantly differ from the respective control groups (*p* > 0.05), *E. faecalis* + PS showed a significant decrease in fibroblast counts compared to its respective control (*p* < 0.05). Notably, the *E. coli* + PS group exhibited a significant increase in cell number compared to the positive control (*p* < 0.05) and was also significantly different from the *E. faecalis* control (*p* < 0.001). These findings suggest that CFS obtained from different bacterial strains influences fibroblast counts differently, and PS may modulate these effects in a strain-dependent manner.

**Figure 3:**
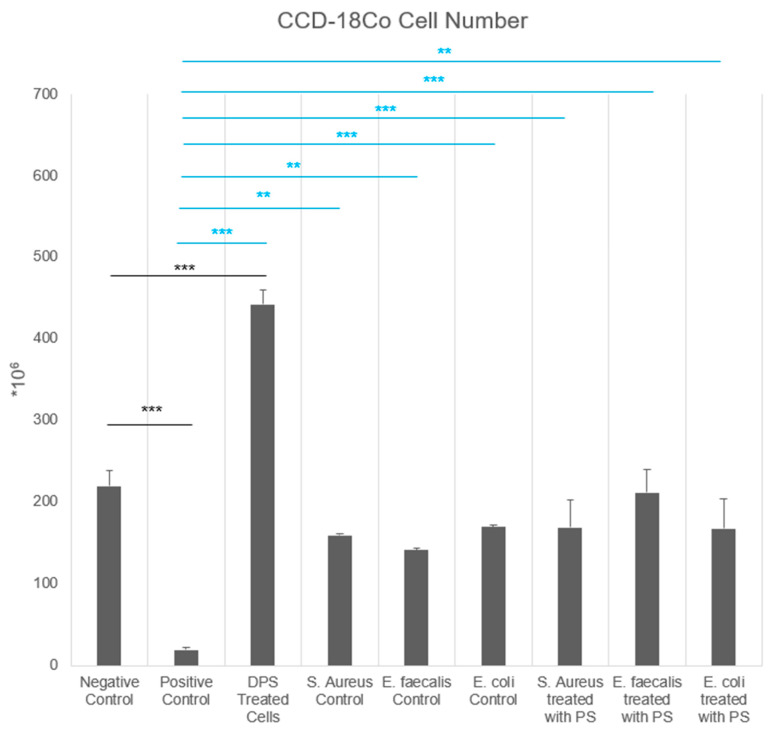
Effect of postbiotic supplement (PS) and bacterial cell-free supernatants (CFS) on CCD-18Co fibroblast cell number. CCD-18Co fibroblast cells were treated with either directly PS (DPS) or CFS obtained from *Staphylococcus aureus*, *Enterococcus faecalis*, and *Escherichia coli* cultured in either standard BHI broth or BHI broth supplemented with PS. Cell numbers were quantified after 48 h of treatment. Data are expressed as mean ± standard deviation (SD). Statistical significance level was determined as *p* < 0.0001. ** *p* < 0.01 and *** *p* < 0.001.

The analysis of CCD-18Co fibroblast cell number revealed significant differences across experimental groups (*p* < 0.0001), indicating strong statistical significance (Figure 3). The positive control group exhibited a substantial reduction in cell number compared to the negative control (*p* < 0.001), demonstrating the strong inhibitory effect of H_2_O_2_. In contrast, the number of DPS showed the highest cell count among the PS groups (*p* < 0.001), demonstrating a boosting effect of PS on CCD-18Co cells. The *S. aureus*, *E. faecalis*, and *E. coli* control groups showed comparable cell numbers with the negative control group, but still significantly lower than DPS-treated cells (*p* < 0.001). The *S. aureus* + PS, *E. faecalis* + PS, and *E. coli* + PS groups exhibited cell numbers comparable to the negative control and their respective untreated bacterial CFS controls.

### 3.2. PS and Bacterial CFS Treatments Promote Fibroblast Migration in Scratch Assay

**Figure 4:**
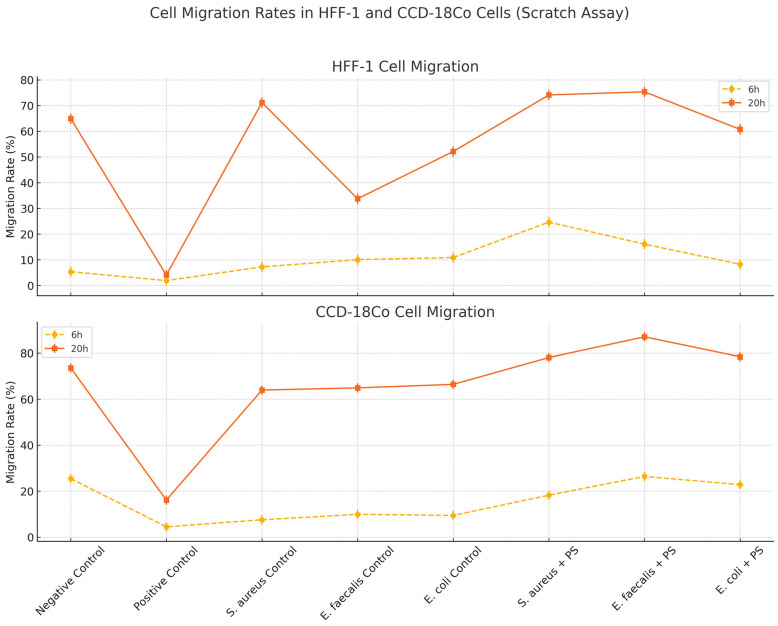
The migration rate (%) of the cell scratch assay in HFF-1 and CCD-18Co cell groups treated with postbiotic supplement.

The cell migration rates of HFF-1 cells at both 6 h and 20 h were significantly influenced by bacterial CFS and PS treatment (*p* = 0.0009 at 6 h, *p* < 0.0001 at 20 h) (Figure 4). At the sixth hour, the positive control group exhibited the lowest migration rate (1.89 ± 0.91%) compared to the negative control (5.34 ± 1.94%, *p* < 0.01, a), indicating that the applied treatment in this group significantly suppressed migration of HFF-1 cells. The *S. aureus*, *E. faecalis*, and *E. coli* control groups showed slightly increased migration rates (7.23–10.87%), but these differences were not statistically significant when compared to the negative control. Notably, the *S. aureus* + PS group exhibited the highest migration rate (24.65 ± 2.93%, *p* < 0.001 vs. negative control, *p* < 0.01 vs. positive control, b, c, e), followed by the *E. faecalis* + PS group (16.08 ± 5.10%, *p* < 0.05, d), indicating that PS significantly enhanced fibroblast migration, particularly in the *S. aureus* + PS group. At the 20th hour, a similar trend was observed for HFF-1 cells. The positive control group remained the lowest (4.04 ± 3.41%) and was significantly lower than the negative control (64.94 ± 13.43%, *p* < 0.001, a, f). Among the untreated bacterial CFS groups, the *S. aureus* control had the highest migration rate (71.18 ± 6.72%) compared to *E. faecalis* control (33.74 ± 6.91%, *p* < 0.01, g) and *E. coli* control (52.13 ± 12.12%). The *S. aureus* + PS (74.14 ± 11.97%) and *E. faecalis* + PS (75.35 ± 9.16%) groups exhibited the highest migration rates at the 20th hour, significantly higher than the *E. faecalis* control group (*p* < 0.001, h) (Figure 4), suggesting that PS enhances fibroblast migration in a strain-dependent manner.

The migration rates of CCD-18Co fibroblast cells were assessed at 6 h and 20 h, and significant differences were observed across groups, particularly at the 20th hour (*p* < 0.0001) (Figure 4). At the sixth hour, the positive control group exhibited the lowest migration rate (4.46 ± 5.13%) compared to the negative control (25.46 ± 12.28%), indicating a suppressive effect. The *S. aureus*, *E. faecalis*, and *E. coli* control groups showed moderate migration rates ranging from 7.54% to 9.89%, with no significant difference from the negative control (*p* > 0.05). The highest migration rate at this time point was observed in the *E. faecalis* + PS group (26.39 ± 13.33%), followed by the *E. coli* + PS group (22.81 ± 19.70%) and the *S. aureus* + PS group (18.22 ± 5.65%), suggesting that PS treatment modestly enhanced early fibroblast migration. At the 20th hour, a clear difference emerged between the groups of CCD-18Co cells. The positive control group maintained the lowest migration rate (16.11 ± 3.76%) and was significantly lower than the negative control (73.62 ± 14.70%, *p* < 0.001, f). The *S. aureus*, *E. faecalis*, and *E. coli* control groups showed higher migration rates (63.95 ± 5.56%, 64.92 ± 3.84%, and 66.43 ± 7.02%, respectively), with no significant difference from the negative control (*p* > 0.05). The PS-treated groups exhibited the highest migration rates at the 20th hour. The *E. faecalis* + PS group showed the greatest increase in migration (87.05 ± 5.97%, *p* < 0.001 vs. *E. faecalis* control), followed by the *E. coli* + PS group (78.41 ± 13.02%) and the *S. aureus* + PS group (78.08 ± 12.32%).

### 3.3. Modulation of Oxidative Stress by PS and Bacterial CFS in Fibroblasts

Analysis of TOS levels in HFF-1 cells showed that direct PB treatment did not change TOS levels (0.45 ± 0.18 µmol H_2_O_2_ equiv/L) compared to those of the negative control group (0.88 ± 0.86 µmol H_2_O_2_ equiv/L) (*p* = 0.700). Similarly, analysis of TAS levels in HFF-1 cells showed that direct PB treatment did not change TAS levels (0.15 ± 0.03 mmol Trolox equiv/L) compared to those of the negative control group (1.38 ± 0.01 mmol Trolox equiv/L) (*p* = 0.333). Comparing the CFS groups of HFF-1 cells, TOS levels significantly varied among the experimental groups (*p* = 0.0119), indicating that bacterial CFS and PS treatments influenced oxidative stress. The positive control group exhibited the highest TOS level (4.57 ± 0.59 µmol H_2_O_2_ equiv/L, *p* < 0.01 vs. negative control), suggesting increased oxidative stress. Among the bacterial CFS-treated groups, the *E. coli* control had the highest TOS level (5.22 ± 1.71 µmol H_2_O_2_ equiv/L, *p* < 0.01 vs. negative control), followed by the *S. aureus* control (3.79 ± 0.44 µmol H_2_O_2_ equiv/L, *p* < 0.05) and the *E. faecalis* control (2.79 ± 0.88 µmol H_2_O_2_ equiv/L). PS treatment significantly reduced oxidative stress. The *S. aureus* + PS and *E. faecalis* + PS groups exhibited the lowest TOS levels (2.06 ± 0.53 and 2.00 ± 0.36 µmol H_2_O_2_ equiv/L, respectively), while the *E. coli* + PS group showed a moderate decrease (2.59 ± 0.32 µmol H_2_O_2_ equiv/L). For total antioxidant status (TAS), no significant differences were observed between groups (*p* = 0.6867). However, the *E. faecalis* + PS group exhibited the highest TAS value (0.93 ± 0.45 mmol Trolox equiv/L), suggesting a potential antioxidant-enhancing effect (Table 2).

In CCD-18Co cells, the TOS levels significantly differed between groups (*p* < 0.0001), demonstrating oxidative stress modulation by bacterial CFS and PS. The positive control group displayed the highest oxidative stress (5.48 ± 1.43 µmol H_2_O_2_ equiv/L, *p* < 0.01 vs. negative control). The *E. coli* control also exhibited high oxidative stress (5.28 ± 1.12 µmol H_2_O_2_ equiv/L, *p* < 0.01 vs. negative control), followed by the *S. aureus* control (1.01 ± 1.28 µmol H_2_O_2_ equiv/L) and *E. faecalis* control (1.96 ± 0.56 µmol H_2_O_2_ equiv/L, *p* < 0.05). PS significantly reduced oxidative stress. The *S. aureus* + PS and *E. faecalis* + PS groups exhibited the lowest TOS levels (0.45 ± 0.23 and 0.48 ± 0.15 µmol H_2_O_2_ equiv/L, respectively, *p* < 0.01 vs. positive control). The *E. coli* + PS group showed a partial reduction in oxidative stress (1.04 ± 0.62 µmol H_2_O_2_ equiv/L) but remained significantly different from the *E. coli* control (*p* < 0.01).

For TAS levels, differences between groups were not statistically significant (*p* = 0.0902), but notable trends were observed. The *E. faecalis* + PS group exhibited the highest TAS level (1.32 ± 0.22 mmol Trolox equiv/L). The *E. coli* + PS group showed a substantial increase in TAS (3.66 ± 2.39 mmol Trolox equiv/L, *p* < 0.01 vs. *E. coli* control), suggesting a potential antioxidant-boosting effect.

### 3.4. Gene Expression Responses to PS and Bacterial CFS in Fibroblast Cells

**Figure 5:**
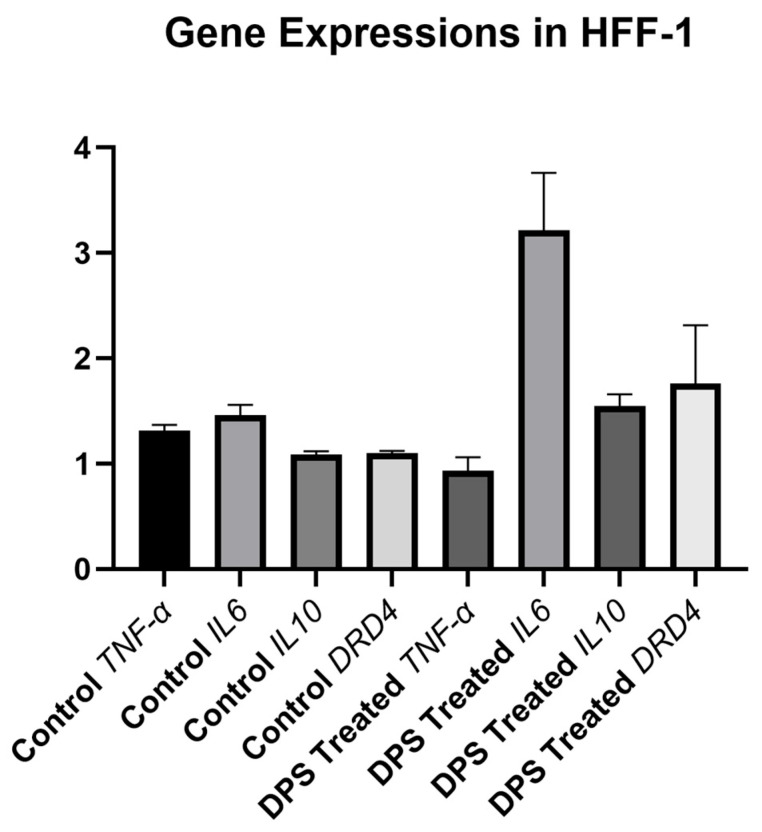
Gene expression levels in HFF-1 cells treated directly with postbiotic supplement (DPS). All gene expressions were measured and normalized to the negative control group. Data are presented as mean ± standard deviation (SD). Statistical analysis indicated no significant differences between groups (*p* = 0.333 for all expressions compared between control and DPS-treated groups).

Analysis of *TNF-α*, *IL-6*, *IL-10*, and *DRD4* gene expressions between the negative control and DPS groups of HFF-1 cells showed no statistically significant differences (*p* = 0.333), indicating that DPS treatment did not alter the gene expressions of *IL-6*, *IL-10*, and *DRD4* genes in HFF-1 cells. *TNF-α* gene showed a relative decrease compared to the control group, but this was not significant (Figure 5).

**Figure 6:**
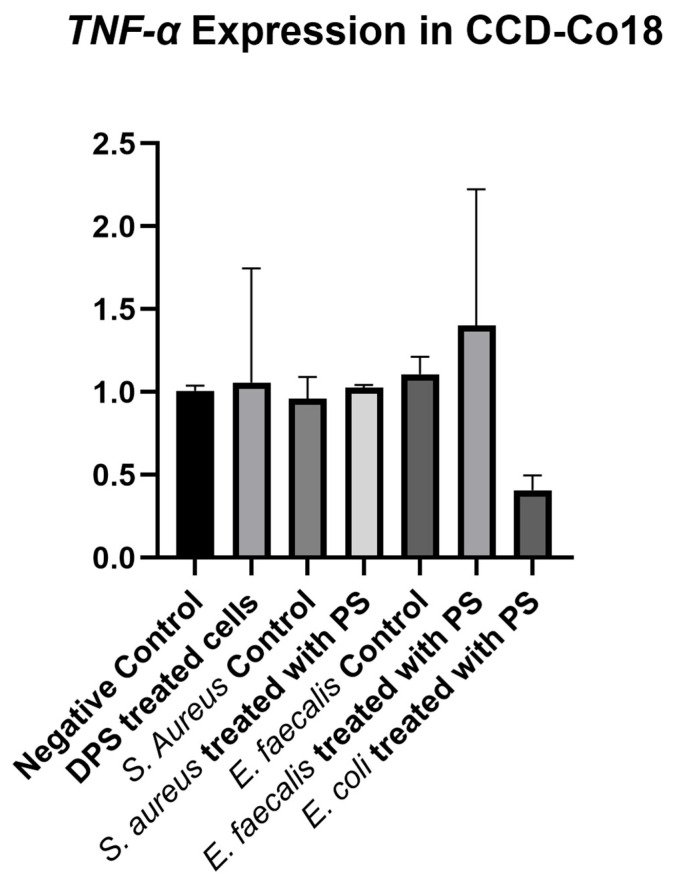
*TNF-α* expression levels in fibroblast cells treated with bacterial cell-free supernatants (CFS) and postbiotic supplement (PS). Fibroblast cells were treated with CFS obtained from *Staphylococcus aureus*, *Enterococcus faecalis*, and *Escherichia coli* cultured in either standard BHI broth or BHI broth supplemented with PS. *TNF-α* expression was measured and normalized to the negative control group. Data are presented as mean ± standard deviation (SD). Statistical analysis indicated no significant differences between groups (*p* = 0.459).

The analysis of *TNF-α* expression across different experimental groups of CCD-18Co cells showed no statistically significant differences (*p* = 0.459), indicating that neither bacterial CFS nor PS treatment had a substantial impact on *TNF-α* levels in fibroblast cells (Figure 6). The negative control group exhibited a baseline *TNF-α* expression level. The Only PS-treated cells showed a slight increase in *TNF-α* expression, but with high variability. The *S. aureus* control and *S. aureus* + PS groups had *TNF-α* expression levels similar to the negative control, suggesting that *S. aureus*-derived CFS had a minimal effect on *TNF-α* regulation. Similarly, the *E. faecalis* control group showed a moderate increase in *TNF-α* levels, whereas the *E. faecalis* + PS group exhibited higher variability with a trend toward increased *TNF-α* expression. The *E. coli* + PS group displayed the lowest *TNF-α* expression level among all groups, suggesting a potential suppressive effect of PS-treated *E. coli* CFS on *TNF-α* production, although this difference was not statistically significant.

**Figure 7:**
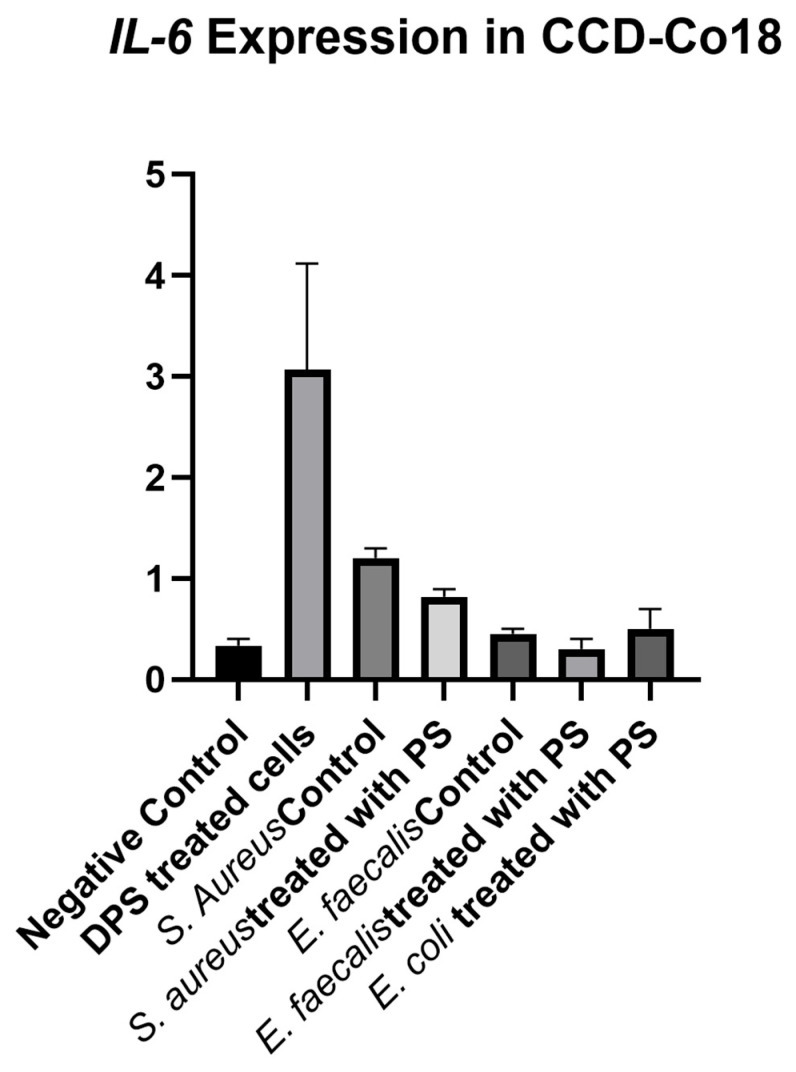
*IL-6* expression levels in fibroblast cells treated with bacterial cell-free supernatants (CFS) and postbiotic supplement (PS). Fibroblast cells were treated with CFS obtained from *Staphylococcus aureus*, *Enterococcus faecalis*, and *Escherichia coli* cultured in either standard BHI broth or BHI broth supplemented with PS. IL-6 expression was measured and normalized to the negative control group. Data are presented as mean ± standard deviation (SD). Statistical analysis indicated no significant differences between groups (*p* = 0.386).

The analysis of *IL-6* expression among the experimental groups of CCD-18Co cells showed no significant differences (*p* = 0.386). In the study, it was concluded that PS treatment relatively increased *IL-6* levels in fibroblast cells, while bacterial CFS treatment showed a limited increase. In bacterial CFS treatment, the highest increase was observed in *S. aureus* bacterial CFS treatment (Figure 7). The negative control group exhibited a baseline *IL-6* expression level that was similar in most experimental groups except for the DPS group. Only PS-treated cells exhibited a noticeable increase in *IL-6* expression, with high variability in measurements. The *S. aureus* control, *S. aureus* + PS, *E. faecalis* control, *E. faecalis* + PS, and *E. coli* + PS groups exhibited *IL-6* expression levels comparable to the negative control, indicating that, relative to bacterial CFS, the directly PS-treated groups induced pro-inflammatory *IL-6* responses under the conditions tested.

**Figure 8:**
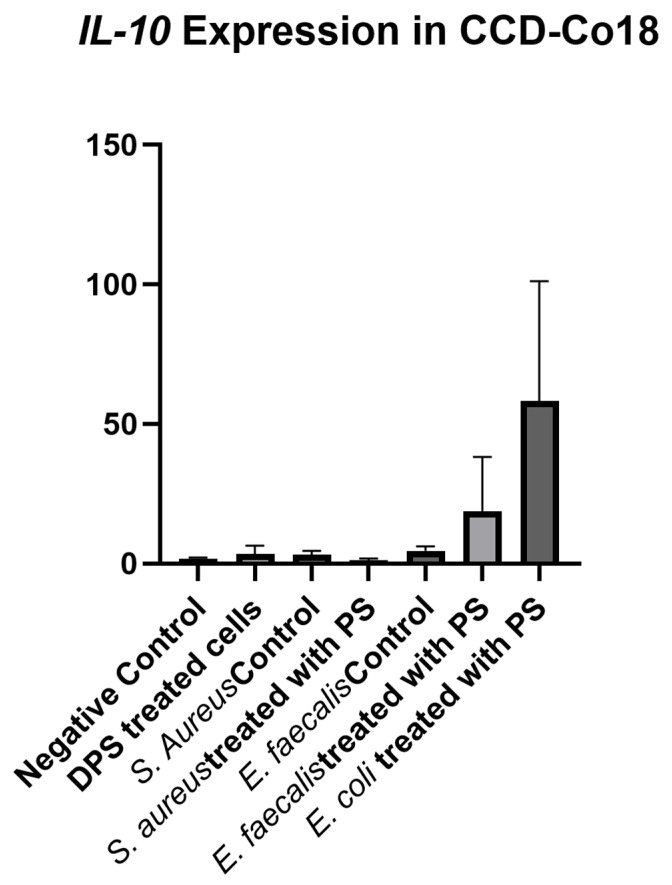
*IL-10* expression levels in fibroblast cells treated with bacterial cell-free supernatants (CFS) and Postbiotic Supplement (PS). Fibroblast cells were treated with CFS obtained from *Staphylococcus aureus*, *Enterococcus faecalis*, and *Escherichia coli* cultured in either standard BHI broth or BHI broth supplemented with PS. *IL-10* expression was measured and normalized to the negative control group. Data are presented as mean ± standard deviation (SD). Statistical analysis indicated no significant differences between groups (*p* = 0.147).

Analysis of *IL-10* expression between the experimental groups of CCD-18Co cells showed no significant difference (*p* = 0.147), indicating that neither bacterial CFS nor PS treatment had a significant effect on *IL-10* levels in fibroblast cells under the conditions tested (Figure 8). The negative control group exhibited increased IL-10 expression, which was consistent across most groups. Cells treated with PS alone, *S. aureus* control, or *S. aureus* + PS and *E. faecalis* control groups exhibited *IL-10* expression levels comparable to the negative control, indicating that neither bacterial CFS nor PS alone affected *IL-10* production in fibroblast cells. The *E. faecalis* + PS group showed a moderate increase in *IL-10* expression, but this increase was not significant. Notably, the *E. coli* + PS group exhibited the highest *IL-10* expression with a large standard deviation, suggesting a potential trend towards increased *IL-10* expression.

**Figure 9:**
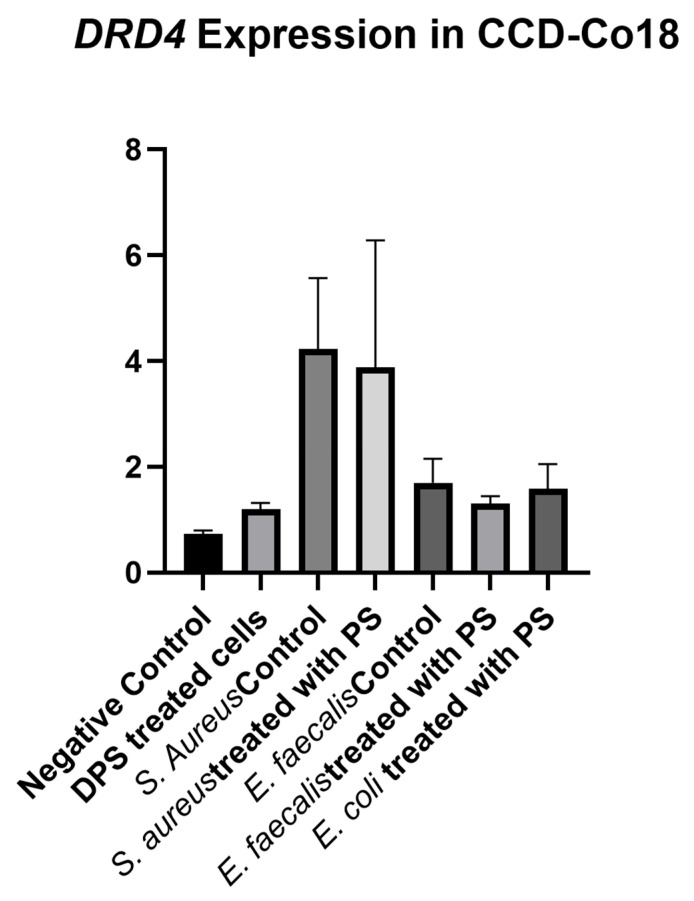
*DRD4* expression levels in fibroblast cells treated with bacterial cell-free supernatants (CFS) and postbiotic supplement (PS). Fibroblast cells were treated with CFS obtained from *Staphylococcus aureus*, *Enterococcus faecalis*, and *Escherichia coli* cultured in either standard BHI broth or BHI broth supplemented with PS. DRD4 expression was measured and normalized to the negative control group. Data are presented as mean ± standard deviation (SD). Statistical analysis indicated no significant differences between groups (*p* = 0.238).

Analysis of *DRD4* expression between experimental groups of CCD-18Co cells showed no significant differences (*p* = 0.238), indicating that neither bacterial CFS nor PS treatment had a significant effect on *DRD4* gene expression in fibroblast cells under the conditions tested (Figure 9). The negative control group exhibited an initial *DRD4* expression level that was approximately similar to that in cells treated with PS alone, indicating that PS alone did not significantly affect *DRD4* expression. *S. aureus* control and *S. aureus* + PS groups exhibited the highest *DRD4* expression levels among all groups. The *E. faecalis* control, *E. faecalis* + PS and *E. coli* + PS groups showed less *DRD4* expression increase than the negative control, suggesting that they were minimally affected by these treatments.

## 4. Discussion

The findings of this study suggest a potential enhancement of fibroblast counts and migration by bacterial CFS, particularly when supplemented with PS, based on trends observed in improved cell counts and scratch assay findings. The most pronounced effects were observed in the *S. aureus* + PS and *E. faecalis* + PS groups, which significantly promoted fibroblast motility, suggesting that PS-modified CFS may promote fibroblast functions relevant to tissue repair, such as migration and oxidative stress modulation. These results are in line with previous studies demonstrating that paraprobiotics and their metabolites can accelerate wound closure by stimulating fibroblast migration and extracellular matrix remodeling [16,17,18]. Similarly, Michels et al. (2023) showed that paraprobiotic-treated NIH-3T3 fibroblasts exhibited enhanced re-epithelialization, reinforcing the role of inactivated probiotic components in promoting cell motility and tissue repair [16].

The observed effects on oxidative stress further support the potential protective role of PS in fibroblasts. In this study, *E. coli* CFS significantly increased TOS levels in both HFF-1 and CCD-18Co cells, whereas *S. aureus* and *E. faecalis* had moderate effects. However, PS effectively reduced oxidative stress, particularly in the *S. aureus* + PS and *E. faecalis* + PS groups, suggesting an antioxidant-modulating effect. These findings align with previous research demonstrating that oxidative stress can impair fibroblast function, leading to compromised tissue integrity and delayed wound healing [16,19]. Notably, Michels et al. (2023) found that paraprobiotics modulated oxidative damage markers, including reactive oxygen species (ROS) and lipid peroxidation products, ultimately enhancing cellular resilience to oxidative insults [16].

Although not the main focus of this study, DPS treatment was included to explore potential mechanistic effects. DPS modulated the expression of *TNF-α*, *IL-6*, *IL-10*, and *DRD4* genes in HFF-1 cells. Specifically, *IL-6*, *IL-10*, and *DRD4* were upregulated, while *TNF-α* was downregulated. These findings are in line with previous studies suggesting that paraprobiotics can regulate immune responses by modulating pro- and anti-inflammatory cytokines [4,7,20,21]. The potential link between microbial interaction and dopamine receptor signaling, as suggested by *DRD4* modulation, is a novel aspect that merits further investigation [22,23]. However, as gene expression analysis was not performed across all experimental groups, these findings should be interpreted as preliminary, and future studies should expand on these results with a broader experimental design.

This study also highlights the strain-dependent effects of bacterial CFS on fibroblast behavior. The *E. faecalis* control group exhibited the highest fibroblast counts, whereas *E. coli* and *S. aureus* CFS reduced cell counts. Postbiotic treatment mitigated these effects, with *E. coli* + PS significantly increasing fibroblast counts compared to the positive control. These findings are consistent with previous reports that different bacterial species exert distinct effects on fibroblast activity [1,24]. For instance, Lukic et al. (2017) demonstrated that *L. plantarum* metabolites enhanced fibroblast counts and migration, while *Bifidobacterium* species showed strain-dependent variability [1]. Notably, in our study, PS-supplemented CFS groups exhibited cell numbers comparable to the negative control and their respective untreated bacterial CFS controls, suggesting that postbiotic supplementation may protect fibroblasts by buffering harmful microbial effects and maintaining baseline viability.

Bacteria play crucial roles in various physiological processes, including metabolism, immune modulation, and host–microbe interactions [25]. Many bacterial species produce metabolites such as short-chain fatty acids (SCFAs), exopolysaccharides, and bacteriocins [26], which can modulate inflammation, oxidative stress, and tissue regeneration [27]. In our study, the control bacterial CFS groups showed strain-dependent effects on fibroblasts: *E. faecalis* CFS promoted the cell count, whereas *E. coli* and *S. aureus* CFS suppressed cell counts and increased oxidative stress. These effects were modulated by PS, suggesting that postbiotics may selectively influence the bioactivity of bacterial secretomes, enhancing fibroblast function and activity.

Although the postbiotic formulation used in this study is based on heat-inactivated bacterial strains, we did not perform direct bacterial viability assays following treatment. Therefore, we acknowledge this as a limitation. Future studies should confirm the inactivation status of bacterial cells—such as through colony-forming unit (CFU) assays—to further validate the non-viability of PS-treated bacteria. It should be noted that any proliferation inhibitors (e.g., mitomycin C) were not applied during the scratch assay. However, the assay duration was limited (6 h and 20 h), and the statistically significant differences in wound closure between treatment groups suggest that the observed effects were primarily due to differential modulation of migratory capacity. Future studies may benefit from incorporating proliferation-blocking agents to more precisely dissect the migratory response, independent of cell division.

Last limitation of the present study is the lack of metabolomic profiling of the postbiotic and bacterial CFS preparations. While we observed functional effects on fibroblasts, the specific bioactive metabolites responsible for these changes remain uncharacterized. Future studies should incorporate targeted or untargeted metabolomic analysis to better understand the molecular composition and mechanisms underlying the observed cellular responses.

Taken together, our findings support the hypothesis that PS-modified bacterial CFS can influence fibroblast behavior in a strain-specific manner, contributing to reduced oxidative stress and improved migration. While the preliminary gene expression data from DPS-treated cells provide insight into possible mechanisms, the main strength of the study lies in demonstrating the modulatory effects of PS on bacterial CFS. Future research should expand gene expression analyses across all treatment groups and evaluate the in vivo relevance of these findings to better understand their implications for regenerative medicine and host-microbiota interactions.

## Figures and Tables

**Table 1:** The postbiotic components of Kiperin^®^ Postbiotic 210 Billion CFU *Rhamnosus* & *Plantarum* Digestion (700 mg).

Content	Function	% Amount	mg/L Capsule
Heat-inactivated *Lactiplantibacillus plantarum*	Active Ingredient	41.5	228.25
Heat-inactivated *Lacticaseibacillus rhamnosus* *	Active Ingredient	41.5	228.25
Hydroxypropyl methyl cellulose	Capsule	17	93.5
Total		100	550

*: *Lacticaseibacillus rhamnosus* 500 × 10^11^ cfu/g.

**Table 2:** Total oxidative Status (TOS) and total antioxidant status (TAS) levels in HFF-1 and CCD-18Co cell groups treated with postbiotic supplement.

HFF-1	TOS (µmol H_2_O_2_ Equiv/L)	TAS (Mmol Trolox Equiv/L)
Negative control	1.06 ± 0.83	0.69 ± 0.25
Positive control	4.57 ± 0.59 ^a^	0.71 ± 0.01
*S. aureus* control	3.79 ± 0.44 ^a^	0.59 ± 0.03
*E. faecalis* control	2.79 ± 0.88 ^a^	0.56 ± 0.01
*E. coli* control	5.22 ± 1.71 ^a^	0.64 ± 0.19
*S. aureus* treated with PS	2.06 ± 0.53	0.68 ± 0.13
*E. faecalis* treated with PS	2.00 ± 0.36	0.93 ± 0.45
*E. coli* treated with PS	2.59 ± 0.32	0.59 ± 0.01
*p* value	0.0119	0.6867
CCD-18Co	TOS (µmol H_2_O_2_ equiv/L)	TAS (mmol Trolox equiv/L)
Negative control	0.74 ± 0.30	0.12 ± 0.03
Positive control	5.48 ± 1.43 ^b^	0.30 ± 0.19
*S. aureus* control	1.01 ± 1.28 ^c^	0.63 ± 0.003
*E. faecalis* control	1.96 ± 0.56 ^d^	0.65 ± 0.28
*E. coli* control	5.28 ± 1.12 ^b^	0.27 ± 0.18
*S. aureus* treated with PS	0.45 ± 0.23 ^c^	0.76 ± 0.01
*E. faecalis* treated with PS	0.48 ± 0.15 ^c^	1.32 ± 2.02
*E. coli* treated with PS	1.04 ± 0.62 ^c,e^	3.66 ± 2.39 ^a^
*p* value	<0.0001	0.0902

TOS: total oxidant status, TAS: total antioxidant status. ^a^ *p* < 0.05, ^b^ *p* < 0.001 vs. negative control group; ^c^ *p* < 0.001, ^d^ *p* < 0.01 vs. positive control group; ^e^ *p* < 0.001 vs. E. control group.

## Data Availability

The data and materials obtained and analyzed in this study are available from the corresponding author upon reasonable request.
